# Citrus Peel Flavonoid Extracts: Health-Beneficial Bioactivities and Regulation of Intestinal Microecology *in vitro*

**DOI:** 10.3389/fnut.2022.888745

**Published:** 2022-05-24

**Authors:** Ping Li, Xu Yao, Qingqing Zhou, Xia Meng, Tao Zhou, Qing Gu

**Affiliations:** Key Laboratory for Food Microbial Technology of Zhejiang Province, College of Food Science and Biotechnology, Zhejiang Gongshang University, Hangzhou, China

**Keywords:** citrus peel, flavonoid, bioactivity, intestinal microbiota, short-chain fatty acids

## Abstract

Citrus peel and its extracts are rich in flavonoids, which are beneficial to human health. In this study, the extraction, component analysis, biological activity and intestinal microbiota regulation of citrus peel flavonoid extracts (CPFEs) were investigated. CPFEs from 14 Chinese cultivars were purified by ultrasound-assisted extraction and XAD-16 macroporous resin. The total flavonoid content of lemon was greatest at 103.48 ± 0.68 mg/g dry weight (DW) by NaNO_2_-Al(NO_3_)_3_-NaOH spectrophotometry. Using high-performance liquid chromatography–diode array detection, the highest concentrations of naringin, hesperidin and eriocitrin were found in grapefruit (52.03 ± 0.51 mg/g DW), chachiensis (43.02 ± 0.37 mg/g DW) and lemon (27.72 ± 0.47 mg/g DW), respectively. Nobiletin was the most polymethoxylflavone in chachiensis at 16.91 ± 0.14 mg/g DW. CPFEs from chachiensis and grapefruit had better antioxidant activity, α-glucosidase inhibitory and sodium glycocholate binding ability. In addition, chachiensis and grapefruit CPFEs had positive effects on intestinal microecology, as evidenced by a significant increase in the relative abundance of *Bifidobacterium* spp., and production of short-chain fatty acids, especially acetic acid, by a simulated human intestinal model. Collectively, our results highlight the biological function of CPFEs as prebiotic agents, indicating their potential use in food and biomedical applications.

## Introduction

Citrus fruits of the family Rutaceae are popular with consumers around the world, and large numbers are processed industrially. However, a high proportion of waste is generated by industrial citrus processing because of the thick, inedible peel and large inedible seeds. In recent years, citrus by-products have been used in animal feed production or in the extraction of biofunctional components such as pectin, essential oils and flavonoids (1, 2). The development of citrus by-products into high value-added dietary supplements can not only produce functional foods with health benefits but also help to solve the environmental pollution caused by citrus peel landfills and processing wastewater (3).

Citrus peel forms around 40%−50% of the total fruit mass, and is a substantial source of biologically-active substances that enhance health, especially flavonoids (4). The total flavonoid content (TFC) is mainly composed of flavanones and polymethoxylflavones (PMFs), including naringin, hesperidin, narirutin, nobiletin and neohesperidin (5). The most abundant flavonoids vary between different citrus fruits; for example, mandarins and hybrids contain more hesperidin, pummelos contain more naringin, and lemon has the most eriocitrin (6, 7). The major flavonoid from *Citrus unshiu* peel is quercetagetin (8) and anthocyanin is only found in blood oranges (9). Flavonoids in citrus peel are recognized as a good source of dietary antioxidants, and protect cells by hydrogen transfer, free radical scavenging, and divalent metal ion chelation (10). They also help to regulate metabolic syndrome and type 2 diabetes, as manifested by α-glucosidase inhibition, insulin sensitization and decreased blood lipid levels (11). The compositions of flavonoids are closely related to the biological properties. The content of hesperidin in *C. unshiu* peel extracts was positively correlated with the antioxidant activity; hesperetin and naringenin were related to the inhibition of xanthine oxidase and α-glucosidase activities (12).

There is accumulating evidence that dietary flavonoids influence the microbial population of human colon (13). Most dietary flavonoids are poorly absorbed from the small intestine and up to 90% of these compounds are metabolized by the intestinal microflora in the colon (14). Flavonoid and its metabolites interact with the intestinal microflora by inhibiting the growth of pathogenic bacteria and promoting that of beneficial bacteria, and regulate the production of short-chain fatty acids (SCFAs), secondary bile acids and tryptophan metabolites, thereby contributing to maintenance of intestinal homeostasis (15). SCFAs, mainly acetic, propionic and butyric acids, are generated by fermentation of soluble dietary fiber by the gut microbiota, which facilitates nutrient absorption, energy metabolism, maintenance of the intestinal mucosal barrier and strong immunity (15–17). However, limited data suggest that different CPFEs differentially affect gut microbiota composition and abundance, and subsequently alter SCFAs production. Such differences may be related to the main flavonoid components in different CPFEs.

Animal models have been used to study the effects of dietary flavonoids on the intestinal microflora. Supplemental feeding with naringenin (the aglycone of naringin) attenuated colon damage and inflammation symptoms in a dextran sulfate sodium-induced murine model of colitis, suggesting that naringin helps maintain the integrity of the intestinal wall, by protecting the intestinal tight junction barrier (18). Human studies of gut microorganisms *in vivo* are not usually ethically and economically feasible, so *in vitro* simulated human intestinal models have been proposed as an alternative method to study the relationships between the intestinal microbial composition and food components.

In this study, we selected 14 representative citrus cultivars in China and purified CPFEs with ultrasound-assisted extraction and macroporous resin. Quantitatively analysis of 11 components in different CPFEs was performed by high-performance liquid chromatography-diode array detection (HPLC-DAD). We further analyzed the antioxidant, α-glucosidase inhibition and bile salt binding capacity of CPFEs, and their potential effects on microbial composition and SCFAs production were characterized using an *in vitro* simulated human intestinal model.

## Materials and Methods

### Experimental Reagents

Rutin, eriocitrin, naringin, hesperidin, didymin, poncirin, naringenin, hesperitin, sinensetin, nobiletin, tangeretin, α-glucosidase (from *Saccharomyces cerevisiae*), acarbose and 5-O-demethylnobiletin were from Yuanye Bio-Technology Co., Ltd. (Shanghai, China). Tryptone and yeast extract were from Oxoid Co., Ltd. (Basingstoke, UK). Acetic acid, propionic acid, butyric acid, isobutyric acid, valeric acid, and isovaleric acid were from Dr. Ehrenstorfer (Augsburg, Germany). Sodium glycocholate was from Sigma-Aldrich Co., Ltd. (St. Louis, MO, USA). Other reagents and solvents were analytical grade, from Sinopharm Chemical Reagents Co., Ltd. (Shanghai, China).

### Extraction of Flavonoids From Citrus Peel

Fresh citrus fruits were purchased from local suppliers in China (Supplementary Table 1). Flavonoid extracts were prepared by ultrasound-assisted extraction and clean-up on hydrophobic macroporous resin XAD-16, as described previously, with some modifications (19). Citrus peel powder was dried at 40°C for 48 h and then dispersed in 52% ethanol (material-to-liquid ratio of 1 g/42 ml). Ethanol was added to the extract at a final concentration of 80% (v/v) after 17 min of ultrasonic extraction at 325 w. The crude flavonoids were obtained by centrifuging at 8,000 *g* for 15 min after standing at 4°C for 12 h, retaining the supernatant and removing the ethanol by rotary evaporation under 60°C (Buchi R-300, Switzerland). The crude flavonoid extract at a concentration of 107 μg/ml (2 column volumes) was loaded onto the column of macroporous resin XAD-16 at a flow rate of 1.5 ml/min, then eluted with 50% (v/v) ethanol solution (5 column volumes). CPFEs were concentrated by rotary evaporation and then freeze dried (Alpha 1–4 LSC, Martin Christ, Osterode, Germany) for further analysis.

### Citrus Flavonoid Compositions of CPFEs

#### TFC Determination

The TFC of CPFEs were measured using the NaNO_2_/Al(NO_3_)_3_/NaOH spectrophotometric method (20). In brief, 1.00 ml of CPFEs, 0.30 ml of 5% NaNO_2_ (m/v) and 1.00 ml of 60% ethanol (v/v) were added into a volumetric flask and stored at room temperature for 6 min. 0.30 ml of 10% Al(NO_3_)_3_ (m/v) was added and incubated for another 6 min, then 2.00 ml of 1 M NaOH was added. After incubating for 10 min at room temperature, the absorbance was measured at 510 nm by SpectraMax 190 Microplate Reader (Molecular Devices, San Jose, CA). TFC values were expressed as rutin equivalents (mg) per gram DW.

#### Flavonoid Compositions Analysis

Eleven flavonoids were identified in CPFEs by an Agilent 1260 Infinity HPLC system (Agilent Technologies, Santa Clara, CA, USA) coupled with DAD and a Sun FireTM C18 column (4.6 × 150 mm I. D. × 5 μm, Phenomenex, Torrance, CA) at room temperature. The mobile phases consisted of 0.1% acetic acid (A) and acetonitrile (B). The initial phase composition was 15% B, then ramped linearly to 25% B at 5 min, 30% B at 15 min, 40% B at 25 min, 55% B at 35 min, 60% B at 40 min and back to 15% B at 45 min, at a flow rate of 0.7 ml/min. The main flavanones (eriocitrin, naringin, hesperidin, didymin, poncirin, naringenin, and hesperitin) and PMFs (sinensetin, nobiletin, tangeretin, and 5-O-demethylnobiletin) were quantified by comparison with authentic standard solutions at detection wavelengths of 283 and 330 nm, respectively.

### Antioxidant Capacity of CPFEs

Antioxidant capacity was determined by the 2,2-diphenyl-1-picrylhydrazyl (DPPH) free radical scavenging activity, 2,2′-azino-bis (3-ethylbenzthiazoline-6-sulfonic acid; ABTS) free radical scavenging activity, ferric reducing antioxidant power (FRAP), and cupric reducing antioxidant capacity (CUPRAC) methods, and expressed as mg Trolox equivalent (TE) per gram DW (21). The total antioxidant potency composite (APC) index was the average of antioxidant index of above four methods, and calculated as described previously: APC index = (measure score/maximum score) × 100% (20).

#### DPPH Determination

The DPPH free radical scavenging activity was determined as described previously with minor modifications (22). A mixture of DPPH (0.20 mM, 1 ml) and CPFE (5.00 mg/ml, 80 μl) was incubated at 25°C in the dark for 30 min, then the absorbance recorded at 517 nm (SpectraMax 190 Microplate Reader, USA).

#### ABTS Determination

ABTS index was tested by an ABTS method kit (Nanjing Jiancheng Bioengineering Co. Ltd., China). ABTS working solution was prepared with detection buffer, ABTS stock solution and peroxide solution (diluted 40 times with PBS, pH 7) at a ratio of 76:5:4 (v/v/v). Ten microliter of the sample solution (5 mg/ml) was mixed with 170 μl of ABTS working solution and 20 μl of peroxidase (diluted 10 times with detection buffer) thoroughly for 6 min, and the absorbance was recorded at 405 nm.

#### FRAP Determination

Ferric reducing antioxidant power was determined by a total antioxidant capacity assay kit (Nanjing Jiancheng Bioengineering Co. Ltd., China), following the manufacturer's instructions. CPFE (5 mg/ml, 5 μl) was mixed with FRAP radical solution (180 μl) and incubated in the dark for 5 min at 37°C then the absorbance was recorded at 593 nm.

#### CUPRAC Determination

The CUPRAC assay was performed as described previously (23). The assay mixture, of CuCl_2_ (0.01 M, 500 μl), neocuproine (0.075 M, 500 μl), ammonium acetate buffer (1 M, pH 7.0, 500 μl) and CPFE (5 mg/ml, 60 μl) was incubated at 25°C for 1 h, transferred to a 96-well microplate, then the absorbance recorded at 450 nm.

### α-Glucosidase Inhibition Assay

α-Glucosidase inhibition was determined as described previously with some modifications (15). A mixture of CPFE (10 mg/ml, 50 μl) and of α-glucosidase (2 U, pH 6.8, 250 μl) was incubated at 37°C for 10 min. *p*-nitrophenyl glucoside (*p*NPG, 5 mM, 250 μl) was added and incubated for 10 min. Na_2_CO_3_ (0.20 mM, 450 μl) was added to stop the reaction. The reaction mixture was transferred to a 96-well plate (200 μl per well) and the absorbance measured at 405 nm. Acarbose (α-glucosidase inhibitor/diabetes treatment, 12.5 μM) was used as the positive control. The percentage inhibition of α-glucosidase was calculated according to the following formula:


(1) $$\begin{array}{l} \text{α}-\text{glucosidase inhibition }\left(\%\right)\text{ =}\\ \left[\text{1 - }\frac{\left({\text{OD}}_{\text{s}}\text{ - }{\text{OD}}_{\text{b}}\right)\;{\text{-OD}}_{\text{a}}}{{\text{OD}}_{\text{s}}\text{ - }{\text{OD}}_{\text{b}}}\right]\;\times \text{ 100}\%, \end{array}$$

where:


$$\begin{array}{l} \text{ O}{\text{D}}_{\text{s}}:\text{sample}+\text{α}-\text{glucosidase}+\text{pNPG}+\text{O}{\text{D}}_{\text{after reaction}},\\ \text{ O}{\text{D}}_{\text{b}}:\text{sample}+\text{α}-\text{glucosidase}+\text{buffer}+\text{O}{\text{D}}_{\text{after reaction}},\\ \text{O}{\text{D}}_{\text{a}}:\text{acarbose}+\text{α}-\text{glucosidase}+\text{pNPG}+\text{O}{\text{D}}_{\text{after reaction}}. \end{array}$$

### Bile Salt Binding Capacity Determination Assay

Salt binding capacity was calculated by a standard curve with sodium glycocholate as bile acid (24). A mixture of CPFEs (10 mg/ml, 1 ml) and pepsin (10 mg/ml, 1 ml) was incubated in an orbital shaker at 120 rpm and 37°C for 1 h, and then the pH was adjusted to 6.3 with NaOH (0.10 M). Trypsin (10 mg/ml, 4 ml) and salt solutions (1 mM, 4 ml) were added and incubated for 1 h. Precipitated material was removed by centrifugation (4,000 g, 20 min), supernatant (2.5 ml) and H_2_SO_4_ (60% v/v, 7.5 ml) were mixed and heated at 70°C for 25 min, then cooled in an ice bath for 5 min. The reaction mixture was transferred to a 96-well plate and the absorbance was recorded at 387 nm.

### Fecal Sample Collection and Processing

Fecal samples were collected from seven healthy volunteers (three males and four females, numbered 1–7) according to the following criteria: (1) aged from 20 to 35, (2) a body mass index of 18.5–23.9 kg/m^2^, (3) normal diet and not vegetarian, (4) no history of bowel disorders, (5) no antibiotics or probiotics used in the previous 6 months. All donors were provided written informed consent, and the study was approved by the Ethics Committee of the Zhejiang Gongshang University and Zhejiang Academy of Agricultural Sciences (Zhejiang Province, China). Fresh fecal samples were immediately collected, weighed and diluted in anaerobic, sterile phosphate-buffered saline (PBS, pH 7, 0.10 M) to prepare 10% fecal homogenate suspensions (w/v).

### Simulated Intestinal Fermentation *in vitro*

Each culture consisted of sterilized VIS medium (5 ml) (25), fecal suspension (500 μl), and CPFE sample (0.10 g/ml, 500 μl). PBS (500 μl) instead of CPFE solution was used as the blank control. Anaerobic fermentation (10% H_2_, 10% CO_2_ and 80% N_2_) was performed at 37°C in an anaerobic workstation (DG250, Don Whitley Scientific, Bingley, UK). After 24 h, fecal fermentation suspension (1 ml) was collected in an Eppendorf tube for DNA sequencing and SCFA concentration determination.

### DNA Extraction and Sequencing

Genomic DNA was extracted from the fecal fermentation suspension with a QIAamp PowerFecal DNA extraction kit, according to the manufacturer's instructions. 16S rDNA gene high-throughput sequencing of the V3–V4 region was performed by Biomarker Bio-Tech Co., Ltd. (Beijing, China), with an Illumina MiSeq platform. Sequencing primers were 338F (forward primer, ACTCCTACGGGAGGCAGCAG) and 806R (reverse primer, GGACTACHVGGGTWTCTAAT). Analysis was performed using the SILVA database, to assign operational taxonomic units (OTUs) with 97% similarity. Based on the OTU analysis, intestinal microbial richness and diversity were evaluated by Alpha diversity (Ace, Chao, Simpson and Shannon) using the Uparse and Mothur software systems. The bacterial community composition among groups was analyzed at the levels of phylum, class, order, family and genus. Differentially abundant taxa were identified by the linear discriminant analysis (LDA) effect size (LEfSe) with LDA score of 4.0.

### Determination of SCFAs

The contents of acetic, propionic, butyric, isobutyric, valeric and isovaleric acid in fermentation samples were measured by gas chromatography-mass spectrometry (GC–MS) (26). The identification and quantification of chromatographic peaks was achieved by comparison with authentic standards, with crotonic acid (20 mM) as the internal standard. Fermentation medium (500 μl) were mixed with crotonic acid metaphosphate solution (100 μl), and frozen at −30°C for 24 h. The solution was thawed and centrifuged at 8,000 g and 4°C for 3 min, then filtered using a 0.22-μm membrane (Millipore, Burlington, MA). Sample (1 μl) was injected into a GC system fitted with a DB-FFAP GC column (30 m × 0.25 mm I. D. × 0.25 μm, Agilent, China) and H_2_ flame ionization detector. The initial column temperature was 75°C, then increased to 180°C, at 20°C /min and maintained for 1 min, then increased to 220°C at 50°C/min, maintained for 1 min. Both injector and detector temperatures were 250°C. The flow rates of carrier gas N_2_, make-up gas H_2_, and air were 20, 30, and 300 ml/min, respectively.

### Statistical Analysis

All data were expressed as mean ± standard deviation. The significant differences among samples were analyzed using *T*-test, one-way ANOVA and Tukey's test. The value of *P* < 0.05 was considered as statistically significant. Statistical analysis and figuring drawing were carried out using SPSS 22.0 (IBM, Armonk, NY) and GraphPad Prism 8.0 (GraphPad Software Inc., San Diego, CA, USA).

## Results

### TFC and Flavonoid Compositions of CPFEs

The TFC values of 14 CPFEs from different Chinese citrus fruits such as mandarins, oranges, pummelos, kumquats, hybrids and citrons were determined (Table 1). Lemon had the highest TFC (103.48 ± 0.68 mg/g DW), followed by satsuma orange (96.22 ± 0.51 mg/g DW), chachiensis (86.54 ± 0.63 mg/g DW), grapefruit (72.82 ± 1.56 mg/g DW) and fertile orange (67.98 ± 0.86 mg/g DW). Bergamot had the lowest concentration at 27.62 ± 1.25 mg/g DW.

**Table 1:** Contents of total flavonoids, flavanones and PMFs in CPFEs (mg/g DW).

**Cultivar**	**TFC**	**Flavanones**							**PMFs**			
		**Eri**	**Nar**	**Hed**	**Did**	**Pon**	**Nag**	**Het**	**Sin**	**Nob**	**Tan**	**DN**
Satsuma mandarin	96.22 ± 0.51^b^	2.76 ± 0.09^f^	0.19 ± 0.01^i^	41.33 ± 0.21^b^	4.97 ± 0.02^b^	0.42 ± 0.00^c^	ND	0.13 ± 0.00^g^	1.61 ± 0.00^d^	5.90 ± 0.01^b^	3.87 ± 0.01^a^	0.34 ± 0.00^a^
Chachiensis	86.54 ± 0.63^c^	1.93 ± 0.04^g^	0.17 ± 0.03^i^	43.02 ± 0.37^a^	2.55 ± 0.01^d^	0.09 ± 0.00^f^	ND	0.05 ± 0.00^h^	2.31 ± 0.00^b^	16.91 ± 0.14^a^	1.59 ± 0.01^b^	ND
Ponkan	67.17 ± 0.67^e^	0.47 ± 0.01^i^	22.86 ± 0.15^e^	42.05 ± 0.11^b^	0.70 ± 0.01^f^	11.67 ± 0.06^a^	ND	0.29 ± 0.00^c^	0.06 ± 0.00^h^	1.33 ± 0.01^d^	0.28 ± 0.00^e^	0.04 ± 0.00^b^
Lane late navel orange	53.45 ± 0.59^h^	5.71 ± 0.03^d^	4.57 ± 0.03^g^	40.16 ± 0.23^c^	1.24 ± 0.16^e^	0.43 ± 0.01^c^	0.26 ± 0.01^a^	1.37 ± 0.03^a^	1.84 ± 0.02^c^	5.67 ± 0.01^b^	0.73 ± 0.00^c^	0.08 ± 0.00^b^
Blood orange	60.71 ± 0.63^g^	0.96 ± 0.02^h^	2.24 ± 0.16^h^	35.07 ± 0.12^e^	2.55 ± 0.06^d^	0.23 ± 0.07^d^	0.09 ± 0.00^d^	0.07 ± 0.00^h^	0.16 ± 0.00^g^	0.59 ± 0.00^e^	0.17 ± 0.00^f^	0.03 ± 0.00^b^
Apple pomelo	50.60 ± 0.80^i^	1.74 ± 0.53^g^	40.62 ± 0.14^c^	1.77 ± 0.13^h^	0.08 ± 0.01^i^	0.15 ± 0.01^e^	ND	0.20 ± 0.00^e^	ND	0.11 ± 0.00^g^	ND	ND
Majia pomelo	52.27 ± 0.39^h^	2.80 ± 0.03^f^	44.57 ± 0.74^b^	1.84 ± 0.37^h^	0.33 ± 0.01^g^	0.09 ± 0.01^f^	ND	0.05 ± 0.00^h^	0.18 ± 0.00^g^	0.30 ± 0.00^f^	0.02 ± 0.00^g^	ND
Grapefruit	72.82 ± 1.56^d^	4.91 ± 0.11^e^	52.03 ± 0.51^a^	ND	0.07 ± 0.00^i^	0.16 ± 0.01^e^	ND	0.05 ± 0.00^h^	ND	0.04 ± 0.00^h^	ND	ND
Dekopon	62.33 ± 1.07^f^	0.95 ± 0.01^h^	29.56 ± 0.51^d^	39.25 ± 0.91^c^	4.49 ± 0.16^c^	1.23 ± 0.06^c^	0.15 ± 0.00^c^	0.23 ± 0.04^d^	2.66 ± 0.05^a^	1.77 ± 0.01^c^	0.04 ± 0.00^g^	ND
Fertile orange	67.98 ± 0.86^e^	4.78 ± 0.10^e^	10.97 ± 0.45^f^	37.35 ± 0.38^d^	10.50 ± 0.04^a^	0.38 ± 0.09^c^	0.18 ± 0.01^b^	0.07 ± 0.00^h^	0.13 ± 0.01^g^	0.34 ± 0.00^f^	0.04 ± 0.00^g^	ND
Lemon	103.48 ± 0.68^a^	27.72 ± 0.47^a^	0.02 ± 0.00^j^	24.51 ± 0.18^f^	0.19 ± 0.01^h^	0.07 ± 0.00^f^	0.03 ± 0.00^f^	0.08 ± 0.01	0.69 ± 0.00^f^	0.32 ± 0.00^f^	ND	ND
Sichuan kumquat	30.85 ± 0.56^k^	9.29 ± 0.20^b^	1.96 ± 0.08^h^	0.49 ± 0.00^i^	1.52 ± 0.01^e^	ND	0.10 ± 0.00^d^	0.74 ± 0.00^b^	0.87 ± 0.00^e^	0.12 ± 0.00^g^	0.04 ± 0.00^g^	ND
Longyan kumquat	32.47 ± 0.96^j^	5.70 ± 0.92^d^	11.40 ± 0.27^f^	3.11 ± 0.12^g^	0.62 ± 0.02^f^	1.69 ± 0.02^b^	ND	0.18 ± 0.00^f^	0.93 ± 0.00^e^	1.93 ± 0.00^c^	0.38 ± 0.00^d^	0.03 ± 0.00^b^
Bergamot	27.62 ± 1.25^l^	8.55 ± 0.02^c^	0.16 ± 0.00^i^	3.18 ± 0.01^g^	0.17 ± 0.00^h^	0.24 ± 0.00^d^	0.07 ± 0.01^e^	0.08 ± 0.01^h^	ND	0.46 ± 0.00^e^	ND	ND

*TFC, total flavonoid content; Eri, eriocitrin; Nar, naringin; Hed, hesperidin; Did, didymin; Pon, poncirin; Nag, naringenin; Het, hesperitin; Sin, sinensetin; Nob, nobiletin; Tan, tangeretin; DN, 5-O-demethyl-nobiletin.*

*Results were the mean ± SD (n = 3) on a dried weight (g) of citrus peel flavonoid extracts. Flavonoid contents were calculated as mg rutin equivalents (RE)/g DW. ND, not detected. Different letters above the error bars in the same column indicate significant differences among varieties based on Tukey's test (P <0.05)*.

**Figure 1:**
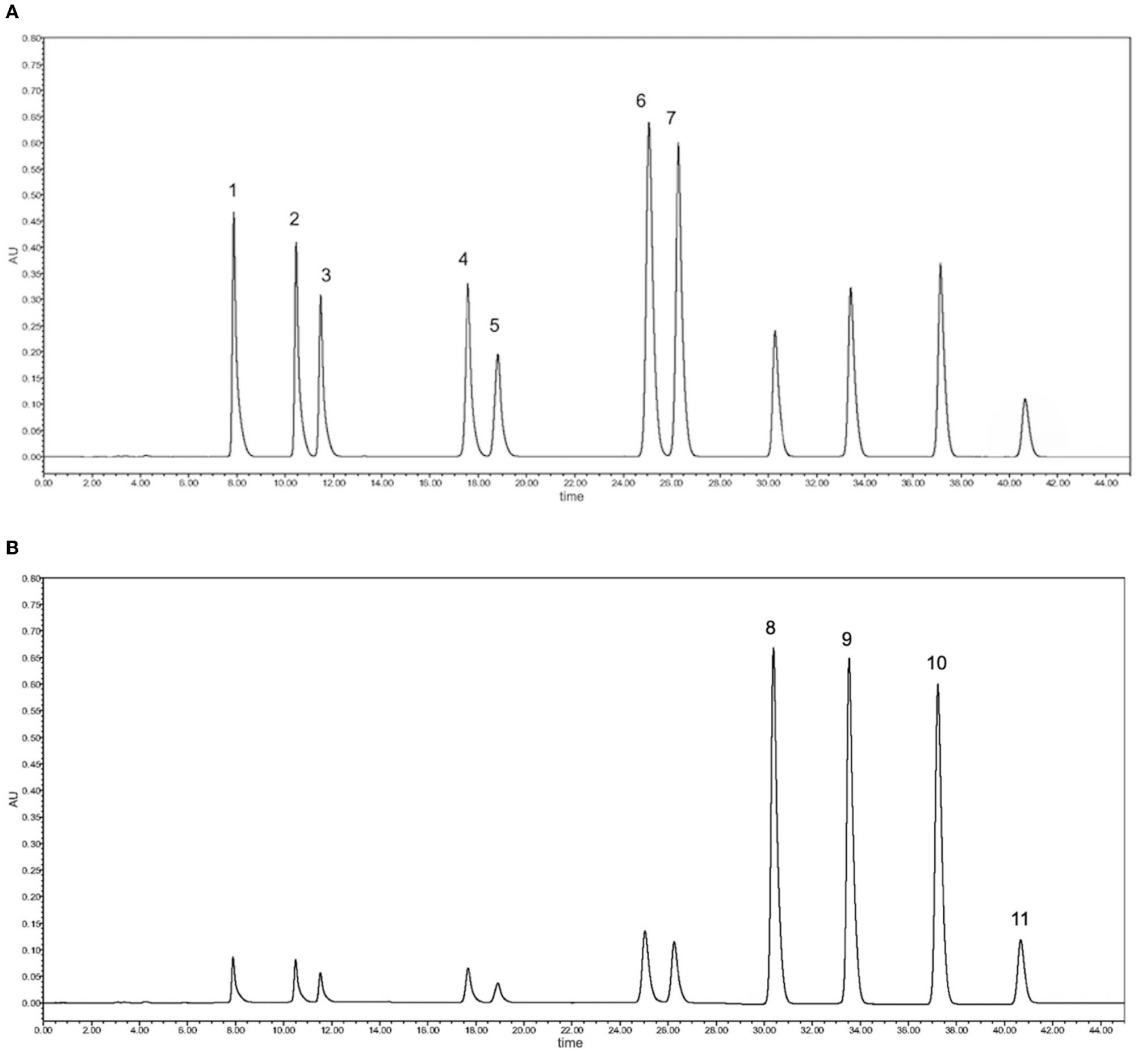
Main flavanones and PMFs were quantified by HPLC-DAD. **(A)** Chromatogram of flavanone standards at 283 nm: 1, eriocitrin; 2, naringin; 3, hesperidin; 4, didymin; 5, poncirin; 6, naringenin; 7, hesperitin. **(B)** Chromatogram of PMF standards at 330 nm: 8, sinensetin; 9, nobiletin; 10, tangeretin; 11, 5-O-demethylnobiletin.

The contents of seven major flavanones (eriocitrin, naringin, hesperidin, didymin, poncirin, naringenin, and hesperitin) and four PMFs (sinensetin, nobiletin, tangeretin, and 5-O-demethylnobiletin) presented significant variation among 14 citrus cultivars (Table 1), calculated by standard curves using HPLC-DAD (Supplementary Table 2). HPLC chromatograms of the standards are shown in Figure 1. Naringin, hesperidin and eriocitrin were the major flavanones, as previously reported (27–29). Abundant naringin was in the CPFEs from grapefruit (52.03 ± 0.51 mg/g DW), majia pomelo (44.57 ± 0.74 mg/g DW), and apple pomelo (40.62 ± 0.14 mg/g DW). However, there was little naringin in the CPFEs from satsuma mandarin, chachiensis, lemon and bergamot. Hesperidin was abundant in the CPFEs from mandarins (satsuma mandarin, chachiensis and ponkan), sweet oranges (lane late navel oranges and blood orange) and hybrids (dekopon and fertile orange), all exceeding 35.00 mg/g DW. In CPFEs from apple pomelo, grapefruit, sichuan kumquat, longyan kumquat and bergamot, the contents of hesperidin were <3.50 mg/g DW. The greatest content of eriocitrin was detected in lemon (27.72 ± 0.47 mg/g DW), and lowest in ponkan (0.47 ± 0.01 mg/g DW). Poncirin was highest in CPFEs (11.67 ± 0.06 mg/g DW), and naringenin was not found in detected mandarin, pummelo and kumquat species.

Compared with flavanones, most CPFEs had lower PMF levels. The contents of sinensetin, nobiletin and tangeretin in chachiensis were the highest at 2.31 ± 0.00 mg/g DW, 16.91 ± 0.14 mg/g DW, and 1.59 ± 0.01 mg/g DW, respectively, followed by satsuma orange (1.61 ± 0.00 mg/g DW, 5.90 ± 0.01 mg/g DW, and 3.87 ± 0.01 mg/g DW) and lane late navel orange (1.84 ± 0.02 mg/g DW, 5.67 ± 0.01 mg/g DW, and 0.73 ± 0.00 mg/g DW). Nobiletin was proved to be the most dominant PMF in mandarins and sweet oranges. The principal PMFs found in grapefruit were tangeretin and nobiletin (27), but our results show that these two PMFs were almost nonexistent. 5-O-Demethylnobiletin was only detected in satsuma mandarin at 0.34 ± 0.00 mg/g DW, but not in most citrus varieties.

### Beneficial Biological Activities of CPFEs

The antioxidant capacity of CPFEs was measured by four separate assays, namely, DPPH, ABTS, FRAP and CUPRAC (Table 2). The DPPH values varied from 17.51 ± 0.34 mg TE/g DW (bergamot) to 55.12 ± 0.08 mg TE/g DW (satsuma orange). There was no significant change in ABTS assay with the other three methods. The highest ABTS radical ability was found in majia pomelo. The CPFEs of mandarins and hybrids presented significantly higher FRAP and CUPRAC values than other cultivars. The overall antioxidant capacity was expressed in APC index, which varied from 39.69 to 92.19%. The top five APC index were classified as grapefruit (92.19%), chachiensis (89.13%), satsuma orange (87.68%), lemon (86.86%) and fertile orange (80.26%), indicating that these CPFEs have better antioxidant properties.

**Table 2:** Antioxidant potency composite index of 14 CPFEs (mg TE/g DW).

**Cultivars**	**DPPH**	**ABTS**	**FRAP**	**CUPRAC**	**APC (%)**	**Rank**
Satsuma orange	55.12 ± 0.08^a^	17.15 ± 0.22^h^	76.25 ± 0.75^c^	25.87 ± 0.27^d^	87.68	3
Chachiensis	43.19 ± 0.11^c^	20.12 ± 0.12^e^	88.84 ± 1.13^a^	25.87 ± 0.17^d^	89.13	2
Ponkan	27.44 ± 0.24^i^	21.37 ± 0.15^b^	79.30 ± 1.11^b^	25.03 ± 0.06^e^	79.99	6
Lane late navel orange	32.67 ± 0.16^g^	21.04 ± 0.16^bc^	53.59 ± 0.43^g^	18.87 ± 0.12^h^	69.51	9
Blood orange	36.03 ± 0.13^e^	20.36 ± 0.06^de^	66.99 ± 1.20^e^	21.02 ± 0.10^g^	75.87	7
Apple pomelo	17.95 ± 0.24^l^	21.42 ± 0.08^b^	25.18 ± 0.60^jk^	8.39 ± 0.05^k^	46.33	12
Majia pomelo	20.18 ± 0.38^k^	22.35 ± 0.11^a^	28.51 ± 0.60^i^	10.76 ± 0.11^i^	51.34	11
Grapefruit	53.73 ± 0.10^b^	21.25 ± 0.06^b^	74.68 ± 0.86^c^	27.04 ± 0.35^c^	92.19	1
Dekopon	34.71 ± 0.11^f^	20.07 ± 0.08^e^	63.75 ± 1.32^f^	34.71 ± 0.11^a^	73.59	8
Fertile orange	37.48 ± 0.18^d^	20.84 ± 0.07^c^	71.15 ± 1.61^d^	23.39 ± 0.05^f^	80.26	5
Lemon	43.16 ± 0.18^c^	18.95 ± 0.24^f^	74.95 ± 0.26^c^	29.35 ± 0.13^b^	86.86	4
Sichuan kumquat	23.96 ± 0.56^j^	17.72 ± 0.11^g^	27.82 ± 0.37^ij^	9.51 ± 0.03^j^	46.62	13
Longyan kumquat	28.94 ± 0.13^h^	20.73 ± 0.12^cd^	35.80 ± 0.80^h^	11.01 ± 0.08^i^	55.77	10
Bergamot	17.51 ± 0.34^l^	15.85 ± 0.09^i^	23.78 ± 0.56^k^	8.60 ± 0.06^k^	39.69	14

*DPPH, free radical scavenging properties by diphenyl-1-picrylhydrazyl radical; ABTS, 2,2′-azino-bis (3-ethylbenzthiazoline-6-sulfonic acid); FRAP, ferric reducing antioxidant capacity; CUPRAC, cupric reducing antioxidant capacity.*

*Data are shown as the mean ± SD (n = 3). The APC index is the average of the antioxidant potency composite index in all tested CPFEs, and APC index = (measured score / maximum score) ×100%. Different letters above the error bars in the same column indicate significant differences among varieties based on Tukey's test (P <0.05)*.

**Figure 2:**
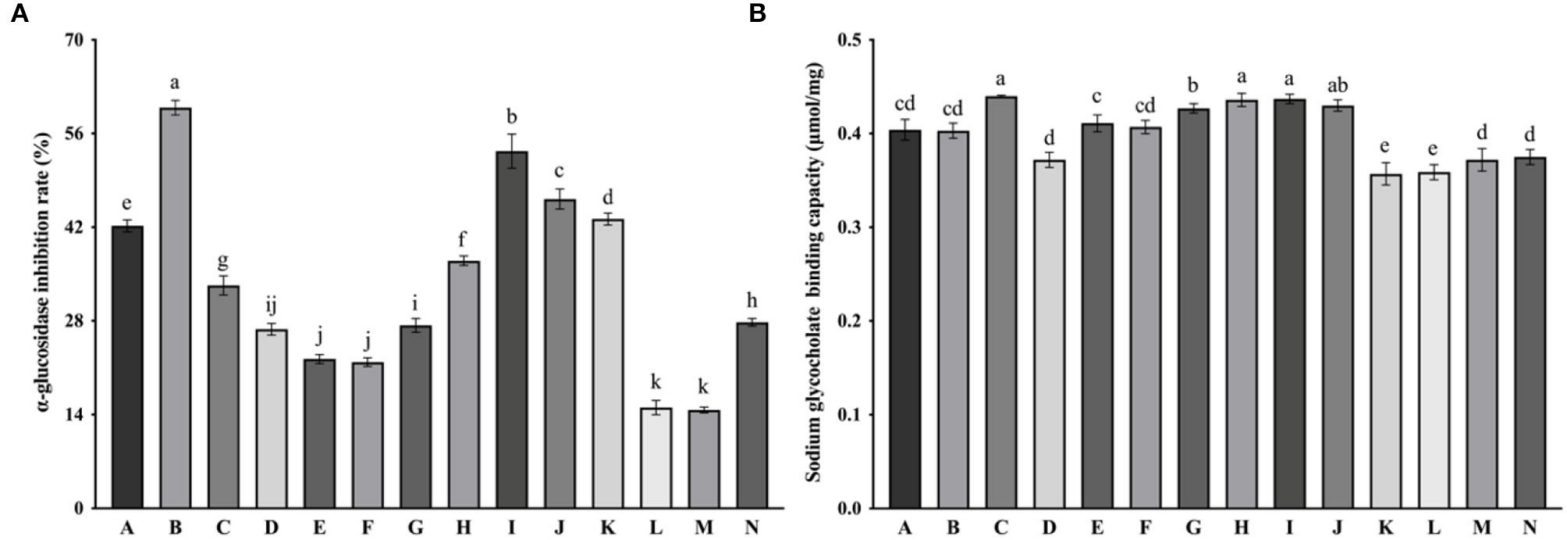
α-Glucosidase inhibition **(A)** and sodium glycocholate binding capacity **(B)** of CPFEs. Data are shown as the mean ± SD (*n* = 3). Different letters the error bars indicate significant differences among varieties based on Tukey's test (*P* < 0.05). A, satsuma orange; B, chachiensis; C, ponkan; D, lane late navel orange; E, blood orange; F, apple pomelo; G, majia pomelo; H, grapefruit; I, dekopon; J, fertile orange; K, lemon; L, sichuan kumquat; M, longyan kumquat; N, bergamot; P, blank control.

Citrus peel flavonoid extracts from mandarins and hybrids had higher inhibitory activity on α-glucosidase than these of sweet oranges, pummelos, kumquats and citrons (Figure 2A). CPFEs with the highest inhibitory effect on α-glucosidase was chachiensis (59.87 ± 1.09%), followed by dekopon (53.38 ± 2.53%), fertile orange (46.21 ± 1.50%), lemon (43.23 ± 0.90%) and satsuma orange (42.21 ± 0.90%). Bergamot, sichuan kumquat and longyan kumquat possessed lower inhibitory activity with values of 27.79 ± 0.57, 15.05 ± 1.06, and 14.69 ± 0.42%, respectively.

The binding capacity of CPFEs to sodium glycocholate ranged from 0.36 ± 0.01 to 0.44 ± 0.00 μmol/mg (Figure 2B). The difference between various CPFEs was not as significant as antioxidant activity and α-glucosidase inhibition. CPFEs from ponkan (0.44 ± 0.00 μmol/mg), dekopon (0.44 ± 0.01 μmol/mg), grapefruit (0.44 ± 0.01 μmol/mg) and majia pomelo (0.43 ± 0.01 μmol/mg) had higher sodium glycocholate binding capacity, suggesting that they have a cholesterol-lowering effect by inhibiting reabsorption of bile acids. However, some eriocitrin-rich citrus such as lemon (0.36 ± 0.01 μmol/mg) and bergamot (0.38 ± 0.01 μmol/mg) were less binding in our test.

### Effects of CPFEs on the Intestinal Microbiota

**Figure 3:**
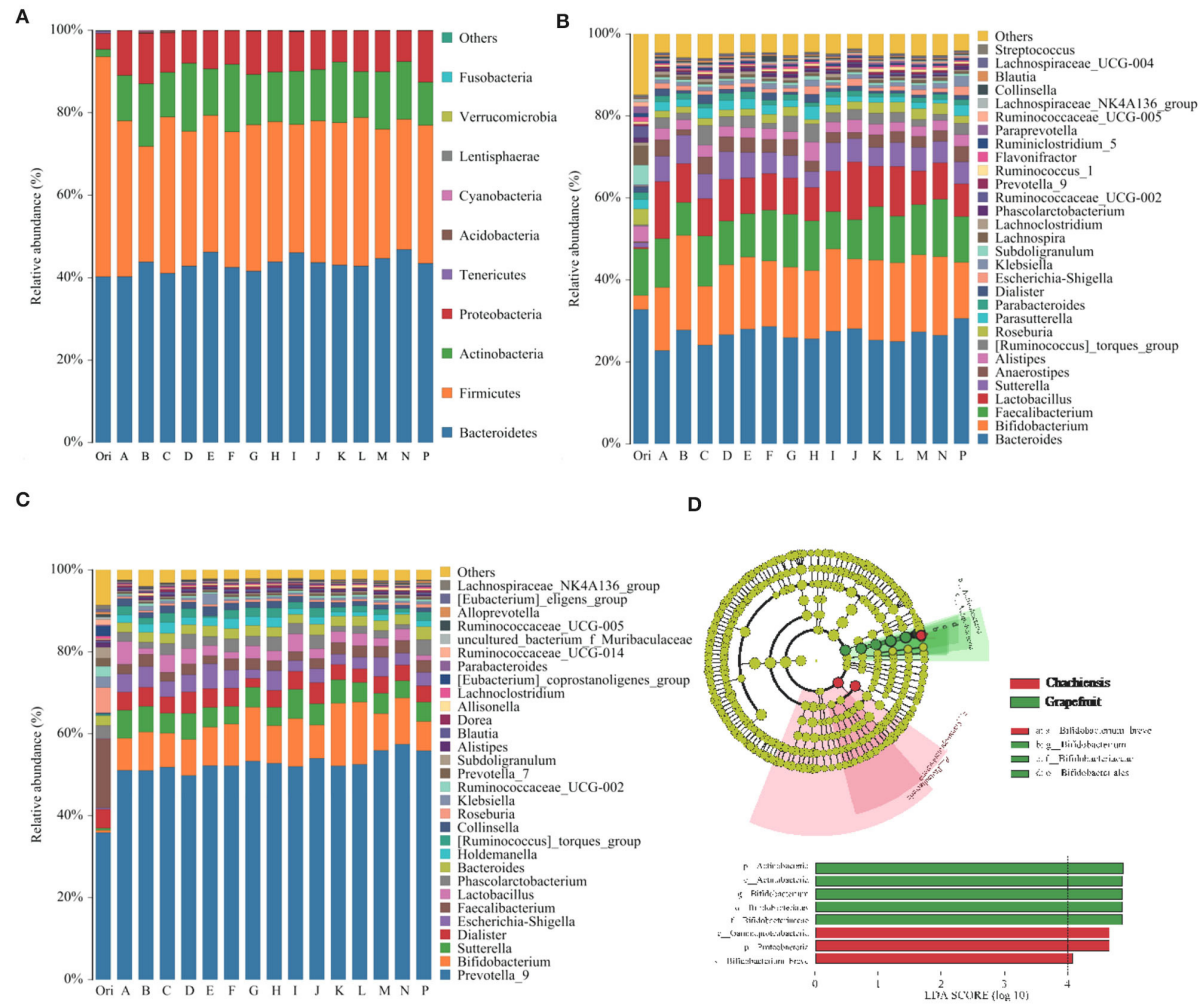
Differences in the distribution and abundance of intestinal microflora in human fecal samples before and after *in vitro* fermentation with CPFEs. **(A)** The compositional changes of intestinal microflora at the phylum level (*n* = 3); **(B)** Test subjects with ET B fecal enterotype at the genus level (subject 1, 2 and 5, *n* = 3); **(C)** Test subjects with ET P fecal enterotype at the genus level (subject 3, 4, 6 and 7, *n* = 4); **(D)** Significant features in microflora of chachiensis and grapefruit CPFEs by LEFSe analysis (LDA > 4.0, *P* < 0.05). A, satsuma orange; B, chachiensis; C, ponkan; D, lane late navel orange; E, blood orange; F, apple pomelo; G, majia pomelo; H, grapefruit; I, dekopon; J, fertile orange; K, lemon; L, sichuan kumquat; M, longyan kumquat; N, bergamot; P, blank control.

Microbial composition and abundance in fecal samples fermentated with different CPFEs were compared using 16S rDNA gene amplicons sequencing. Sequences were classified and assigned to OTUs with more than 97% similarity. Alpha diversity was determined using the Ace, Chao, Shannon and Simpson indices (Supplementary Figure 1). However, there was no significant change in microbial richness and diversity. Microbial composition analysis showed compositional changes at the phylum level (Figure 3A). Bacteroidetes and Firmicutes predominated in the original fecal samples, accounting for more than 90% of the total, but decreased to 70–80% after fermentation with CPFEs. Conversely, the relative abundance of Actinobacter was increased, and the treatment of CPFEs from grapefruit and fertile orange significantly increased Actinobacter abundance to 17.84 and 17.71%, respectively, which only accounted for 10.53% of the blank sample.

At the genus level, the enterotypes of seven volunteers were divided into Enterotype 1 (ET B) and Enterotype 2 (ET P). Subject 1, 2 and 5 belonged to the *Bacteroides*-predominant ET B, and subject 3, 4, 6, and 7 belonged to the *Prevotella*-predominant ET P. The five genera with the highest relative abundance of ET B were *Bacteroides, Bifidobacterium, Faecalibacterium, Lactobacillus* and *Sutterella* (Figure 3B); in ET P, the top five abundances were *Prevotella_9, Bifidobacterium, Sutterella, Dialister* and *Escherichia-Shigella* (Figure 3C). After *in vitro* fermentation with CPFEs, *Bacteroides* became slightly less dominant in the ET B enterotype, decreasing from 30.58 to 26% (average of treatment group), and *Prevotella*_9 became considerably more dominant in ET P. The average relative abundance of beneficial microbial communities *Lactobacillus* and *Bifidobacterium* markedly increased in both enterotypes. Especially in the chachiensis CPFE group, the proportion of *Bifidobacterium* in the ET B group was the highest at 23.09%; and CPFEs of grapefruit and fertile orange increased *Bifidobacterium* to 14.86 and 14.78%, respectively, in the ET P group.

The LefSe analysis highlighted the differences in relative microbial abundance from phylum to species. In all citrus tested, chachiensis and grapefruit were the two cultivars with significant differences in intestinal microbial composition and abundance (Figure 3D). Samples fermented with chachiensis CPFEs had higher levels of the phylum Proteobacteria, class Grammproteobacteria and species *Bifidobacterium breve*. In grapefruit CPFEs, phylum Actinobacteria and genus *Bifidobacterium* were significantly increased. The regulatory effect on intestinal microbiota may be related to the TFC and flavonoid profiles of different CPFEs. Abundant *Bifidobacterium* spp. in the chachiensis group may be associated with high levels of PMFs. Chen et al. found that oral administration of *Citrus reticulata* cv. Suavissima, rich in nobiletin, tangeretin and 5-demethylnobiletin, significantly increased the abundance of the probiotics such as *Bifidobacterium* spp. and *Lactobacillus* spp. (30).

### SCFAs Production and Their Relationship With Microbial Composition

**Figure 4:**
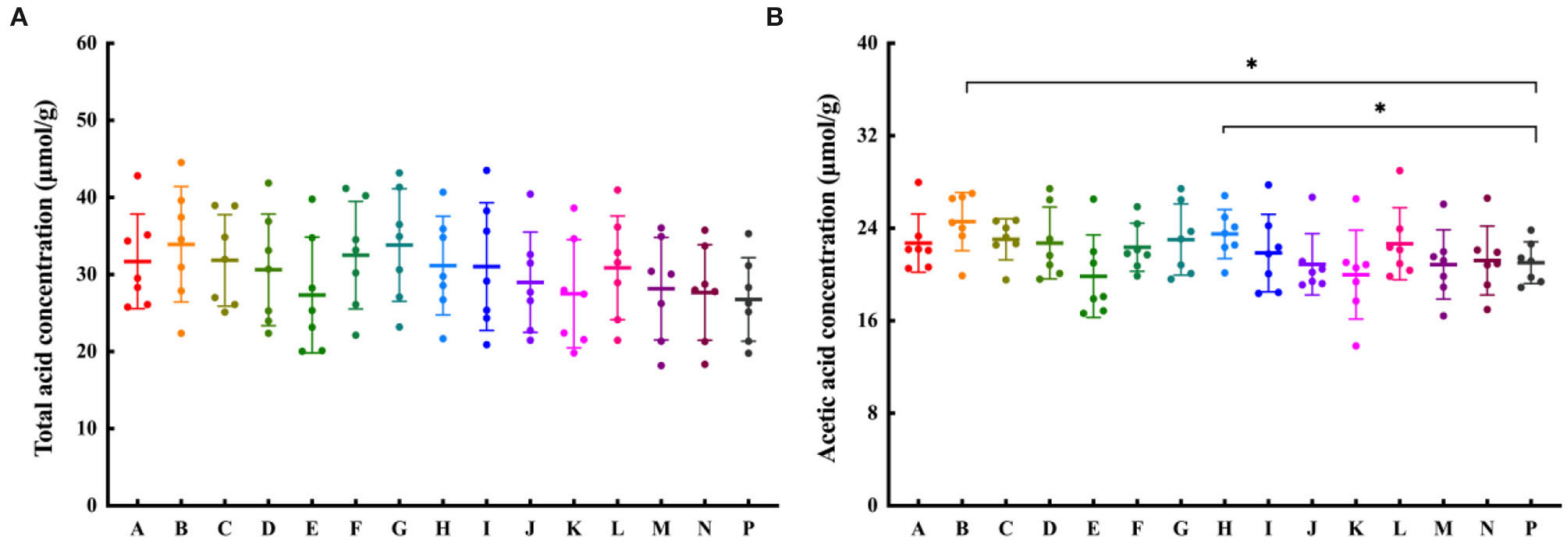
The effect of CPFEs on SCFA production after 24-h *in vitro* fermentation. **(A)** Total SCFA concentration; **(B)** The acetic acid concentration; Data are shown as the mean ± SD (*n* = 7). Significant differences among groups based on *T*-test, * *P* < 0.05. A, satsuma orange; B, chachiensis; C, ponkan; D, lane late navel orange; E, blood orange; F, apple pomelo; G, majia pomelo; H, grapefruit; I, dekopon; J, fertile orange; K, lemon; L, sichuan kumquat; M, longyan kumquat; N, bergamot; P, blank control.

Short-chain fatty acids are the main end-products of indigestible carbohydrate fermentation, and can be used as nutrients by intestinal epithelial cells and the colonic microflora (31). The average levels of total SCFAs generated by fecal microbial fermentations with CPFEs, were all higher than that of the blank control (Figure 4A). Acetic acid was the major SCFA produced, accounting for about 80% of the total (Figure 4B). The concentrations of acetic acid were significantly higher with chachiensis (*P* < 0.05) and grapefruit (*P* < 0.05) compared with the blank control. However, we did not find significant differences in concentrations of butyric, isobutyric, valeric and isovaleric acids (*P* > 0.05, data not shown).

**Figure 5:**
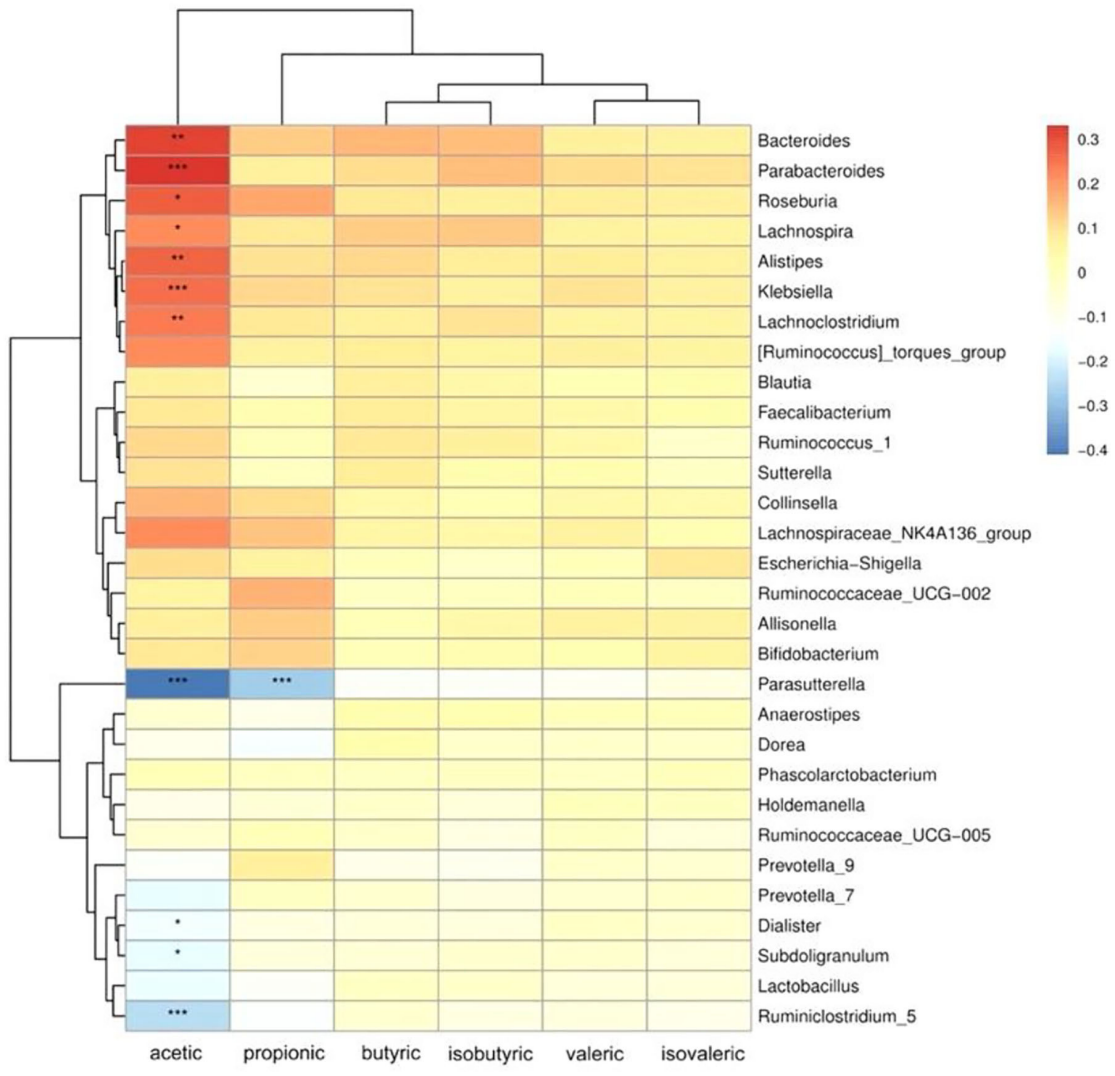
Heatmap of Spearman's rank correlation coefficients between SCFAs production and microbial relative abundance at the genus level. The colors indicate positive (red) or negative (blue) correlations between SCFA production and microbial relative abundance. The *X*-axis shows the different SCFAs, from left to right: acetic acid, propionic acid, butyric acid, isovaleric acid, isobutyric acid, and valeric acid. The *Y*-axis shows different genera. * *P* < 0.05, ** *P* < 0.01, *** *P* < 0.001.

Spearman's correlation analysis was performed to investigate the differences between microbial compositions at the genus level and SCFA productions (Figure 5). Acetic acid was positively correlated with *Bacteroides, Parabacteroides, Roseburia, Lachnospira, Klebsiella, Alistipes* and *Lachnoclostridium*, and negatively correlated with *Parasutterella, Dialister, Subdoligranulum* and *Ruminiclostridium*. We can also find that propionic acid was negatively correlated with *Parasutterella*, and positively correlated with the relative abundance of *Roseburia, Lanospiraceaece_NK4A136, Ruminococcaceae_UCG, Alistipes* and *Bifidobacterium*, but not significantly. The positive relationship between butyric, isobutyric and *Bacteroides, Parabacteroides* and *Lachnospira* was more obvious. However, valeric and isovaleric acids were not significantly associated with gut microbiota.

## Discussion

To the best of our knowledge, this is the first report on the extraction and compositional analysis of flavonoids from the peel of fourteen local Chinese citrus cultivars and their role in regulating the gut microbiota. TFC values of CPFEs in our test were considerably higher than those reported from the same fruits, using ultrasound-assisted extraction alone (4, 23). This appears to be due to the much higher extraction efficiency of macroporous resin XAD-16. Column chromatography with XAD-16 increased the extractable flavonoid content of *Glycyrrhiza glabra* L. leaf from 16.80 to 55.60%, compared with the crude solvent extracts (23). Naringin, hesperidin and eriocitrin are the top three flavanones detected by HPLC-DAD. As previously reported, naringin is rich in hybrids (grapefruit) and pummelos (apple pomelo, majia pomelo); hesperidin is rich in mandarins (satsuma mandarin, chachiensis, ponkan), sweet oranges (lane late navel orange, blood orange), and hybrids (dekopon, fertile orange); and eriocitrin is only enriched in lemon (27–29).

Citrus-derived flavonoids have various human health-promoting functions, such as antioxidant activity, α-glucosidase inhibition and sodium glycocholate binding capacity, which are associated with antihyperglycemic and hyperlipidemic effects (32). Long et al. found that CPFEs with higher content of TFC had stronger antioxidant activities (28). Various *in vitro* and *in vivo* studies have identified that eriocitrin, naringin and hesperidin all have good antioxidant activities, which are beneficial for free radical scavenging, reducing hepatic gluconeogenesis and increasing insulin sensitivity (28, 29, 31, 33). We found that CPFEs with higher APC indices such as grapefruit and chachiensis tend to have higher TFCs. CPFEs from sichuan kumquat, Longyan kumquat and bergamot had lower levels of TFCs and main flavonoids (eriocitrin, naringin, and hesperidin), with poor antioxidant capacity. The richness of naringin and hesperidin in CPFEs can regulate hepatic cholesterol synthesis by inhibiting the activity of 3-hydroxy-3-methylglutaryl-CoA reductase (34, 35). Kwon et al. also found that eriocitrin has cholesterol-lowering properties and inhibits obesity by increasing cellular fatty acid oxidation and energy expenditure, and reducing lipogenesis-related gene expression (36). While CPFEs of apple pomelo and majia pomelo had high content of naringin in our test, their antioxidant activity and α-glucosidase activity were relatively poor. And Zeng et al. showed that hesperidin hydrolysates intensively inhibited α-glucosidase activity whereas hesperidin showed little activity (37). There is limited understanding of the differences in biological activities of various flavonoids. Further analysis on the correlation between the biological activities and main flavonoid components such as hesperidin, naringin and eriocitrin will help the high-value utilization of different varieties of citrus peels and processing wastewater.

Flavonoids derived from citrus peel represent the alterations of gut microbiota. Researchers at the European Molecular Biology Laboratory have proposed the classification of human colonic microbiomes into three “Enterotypes” at the genus level (38). ET B, dominated by genus *Bacteroides*, is associated with high consumption of protein and animal fat. ET P, dominated by genus *Prevotella*, is associated with high carbohydrate consumption. Different enterotypes may be associated with health status and incidence of diseases. Prebiotics and probiotics affect specific bacterial populations in the intestine, which are associated with an individual's enterotype (39). Only Rodríguez-Daza et al. (40) found that supplementation with polyphenol-rich blueberry fruit powders changed the enterotype of obese mice from the Firmicute/*Ruminococcus* enterotype into the healthier *Prevotella*/Akkermansiaceae enterotype. *Bifidobacterium* have been reported to play important roles in regulating intestinal microbiota and mucosal inflammation, contributing to inhibit obesity, diabetes and inflammatory bowel disease (41, 42). *Bifidobacterium* was significantly increased after cofermentation of chachiensis and grapefruit CPFEs with fecal samples (Figure 5), which were associated with the enrichment of naringin and hesperidin, respectively. The effects of naringin and hesperidin on the growth of *Bifidobacterium* strains were dose-dependent (43). In the animal model of high-fat diet, naringin intervention altered the community composition of the gut bacteria, characterized by increased benefits (*Butyricicoccus* etc.) and fewer harmful bacteria (*Campylobacter* etc.) (44).

It is well established that SCFAs are the major components in regulating gut health (45). Dietary citrus flavonoids can alter the abundance of SCFAs in the gut. CPFEs from chachiensis and grapefruit stimulated intestinal acetic acid (Figure 4B). Acetic acid is the main SCFA produced by most fecal bacteria and an important pH regulator in the colon, helping to maintain colonic homeostasis (45). Zhang et al. (46) found that dietary supplements with citrus peel extracts have anti-obesity activity, by increasing the amount of fecal acetic acid by 43% and propionic acid by 86%. After 2 months of drinking pasteurized orange juice containing flavanones, the proportions of total SCFA and acetate were increased in the feces of healthy subjects, and the ammonium concentration was reduced (15, 47). We found that acetate is positively associated with *Roseburia* in the gut (Figure 5). *Roseburia* is a symbiotic beneficial flora that produces SCFAs, affecting colonic motility, immune responses and anti-inflammatory properties (48). Conversely, *Parasutterella* is inversely proportional to acetic and propionic acids. The feces of IBS patients were rich in *Parasutterella*, which was significantly positively correlated with the ratio of inflammatory cells to epithelial cells (49). These suggest that the probiotic effects of citrus flavonoid, if replicated in humans, may confer health benefits.

## Conclusions

In conclusion, CPFEs from 14 Chinese cultivars were extracted and purified, and seven flavanones and four PMFs were quantitatively analyzed by HPLC-DAD. The results of biological function test showed that CPFEs, especially from chachiensis and grapefruit, had good antioxidant activity, α-glucosidase inhibition and bile acid binding capacity. Furthermore, chachiensis and grapefruit CPFEs were found to promote the growth of intestinal *Bifidobacterium* spp. and increase acetic acid content by *in vitro* simulated human gut models. Our results provided valuable insights into understanding the biofunctional activity and gut microbiota regulation of citrus peel flavonoids. Further studies will be performed to investigate the effects of specific flavonoid components such as naringin, hesperidin and eriocitrin on intestinal disease models.

## Data Availability Statement

The datasets presented in this study can be found in online repositories. The names of the repository/repositories and accession number(s) can be found in the article/Supplementary Material.

## Author Contributions

PL and QG: conceptualization and supervision. XY, XM, and QZ: methodology, investigation, and data curation. XY: writing-original draft preparation. QZ and TZ: writing-review and editing. QG: funding acquisition. All authors have read and agreed to the published version of themanuscript.

## Funding

The work was supported by National Key Research and Development Program of China (2017YFE0122300).

## Conflict of Interest

The authors declare that the research was conducted in the absence of any commercial or financial relationships that could be construed as a potential conflict of interest.

## Publisher's Note

All claims expressed in this article are solely those of the authors and do not necessarily represent those of their affiliated organizations, or those of the publisher, the editors and the reviewers. Any product that may be evaluated in this article, or claim that may be made by its manufacturer, is not guaranteed or endorsed by the publisher.
