# Supplementary material for: Citrus Peel Flavonoid Extracts: Health-Beneficial Bioactivities and Regulation of Intestinal Microecology in vitro
Source: Front Nutr. 2022 May 24;9:888745. doi: 10.3389/fnut.2022.888745 (PMC9171401; doi:10.3389/fnut.2022.888745)
Supplement: Supplementary file 1 [file Table_1.DOCX]

Supplementary Material

# Supplementary Tables

**Supplementary Table 1.** Fourteen cultivars of fresh citrus fruits from local suppliers in China.

| Cultivar | Fruit types | Locality (China) |
| --- | --- | --- |
| Satsuma mandarin | Mandarins | Wenzhou City, Zhejiang Province |
| Chachiensis | Mandarins | Xinhui City, Guangdong Province |
| Ponkan | Mandarins | Wenzhou City, Zhejiang Province |
| Lane late navel orange | Sweet oranges | Yichang City, Hubei Province |
| Blood orange | Sweet oranges | Zizhong City, Sichuan Province |
| Apple pomelo | Pummelos | Lixian City, Hunan Province |
| Majia Pomelo | Pummelos | Guangfeng City, Jiangxi Province |
| Grapefruit | Hybrids | Changshan City, Zhejiang Province |
| Dekopon | Hybrids | Pujiang City, Sichuan Province |
| Fertile orange | Hybrids | Wuming City, Guangxi Province |
| Lemon | Hybrids | Anyue City, Sichuan Province |
| Sichuan kumquat | Kumquats | Suichuan City, Jiangxi Province |
| Longyan kumquat | Kumquats | Longyan City, Fujian Province |
| Bergamot | Citrons | Jinhua City, Zhejiang Province |

**Supplementary Table 2.** Standard curves of flavonoid standards by HPLC with DAD detector.

| Standards | Standard curve | R^2^ | Detection wavelength (nm) | Retention time  (min) | Linearity range (μg/mL) | Quantitation limit (μg/mL) |
| --- | --- | --- | --- | --- | --- | --- |
| Eriocitrin | *y* = 10.148 *x* − 34.765 | 0.9994 | 283 | 7.996 | 1.00−500.00 | 0.05 |
| Naringin | *y* = 11.123 *x* − 32.310 | 0.9997 | 283 | 10.487 | 1.00−500.00 | 0.02 |
| Hesperidin | *y* = 9.951 *x* + 11.538 | 0.9991 | 283 | 11.590 | 1.00−500.00 | 0.04 |
| Didymin | *y* = 10.112 *x* − 21.889 | 0.9999 | 283 | 17.686 | 1.00−500.00 | 0.03 |
| Poncirin | *y* = 12.611 *x* − 35.625 | 0.9999 | 283 | 18.890 | 1.00−500.00 | 0.10 |
| Naringenin | *y* = 17.215 *x* − 34.383 | 0.9992 | 283 | 25.118 | 1.00−500.00 | 0.03 |
| Hesperitin | *y* = 18.001 *x* − 43.262 | 0.9999 | 283 | 26.484 | 1.00−500.00 | 0.03 |
| Sinensetin | *y* = 22.135 *x* + 118.72 | 0.9934 | 330 | 30.297 | 1.00−250.00 | 0.03 |
| Nobiletin | *y* = 24.371 *x* − 79.498 | 0.9981 | 330 | 33.641 | 1.00−250.00 | 0.02 |
| Tangeretin | *y* = 29.824 *x* + 44.824 | 0.9994 | 330 | 37.420 | 1.00−250.00 | 0.02 |
| 5-O-demethyl-nobiletin | *y* = 18.671 *x* − 12.792 | 0.9994 | 330 | 40.894 | 1.00−250.00 | 0.04 |

## Supplementary Figures


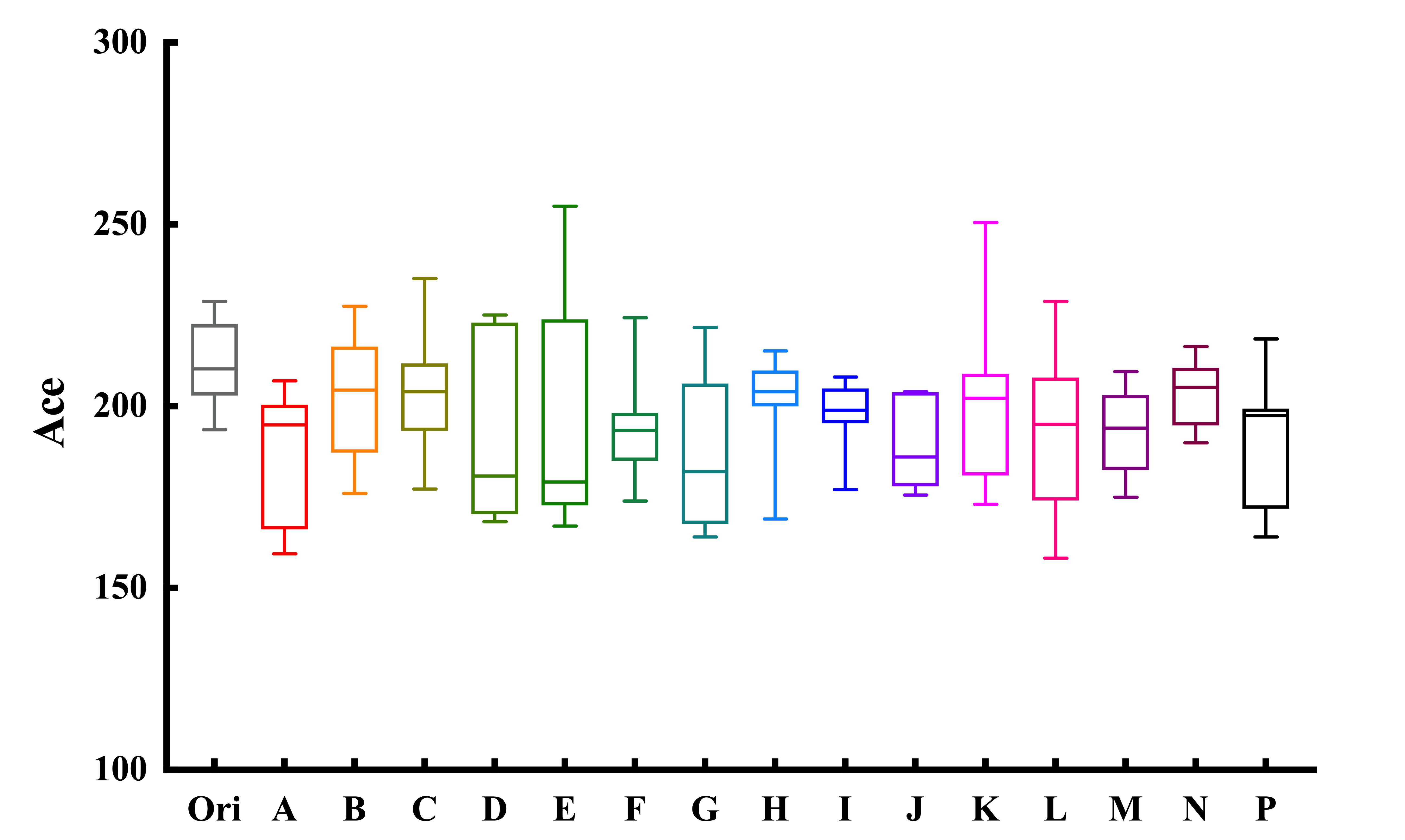

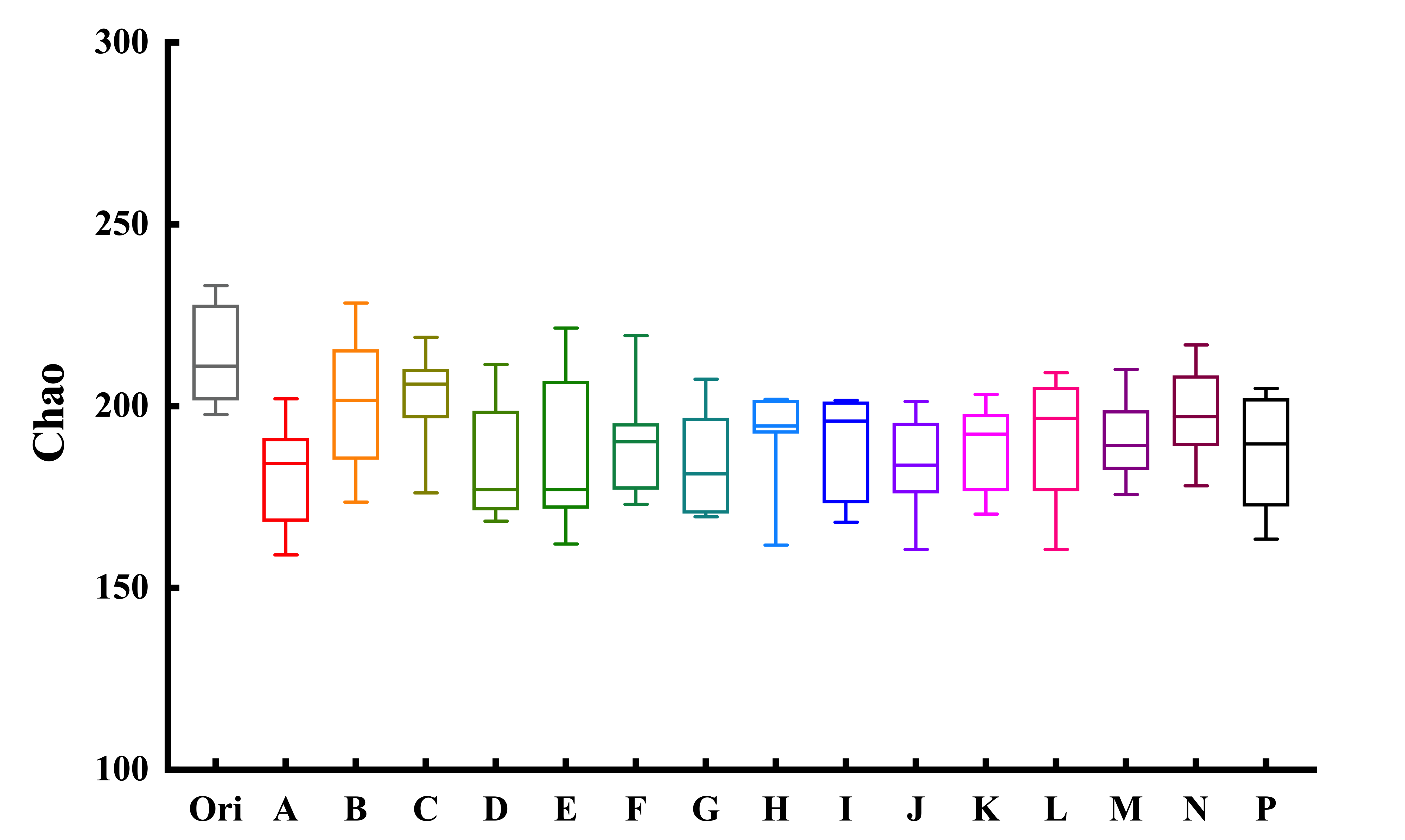


(A) (B)

**
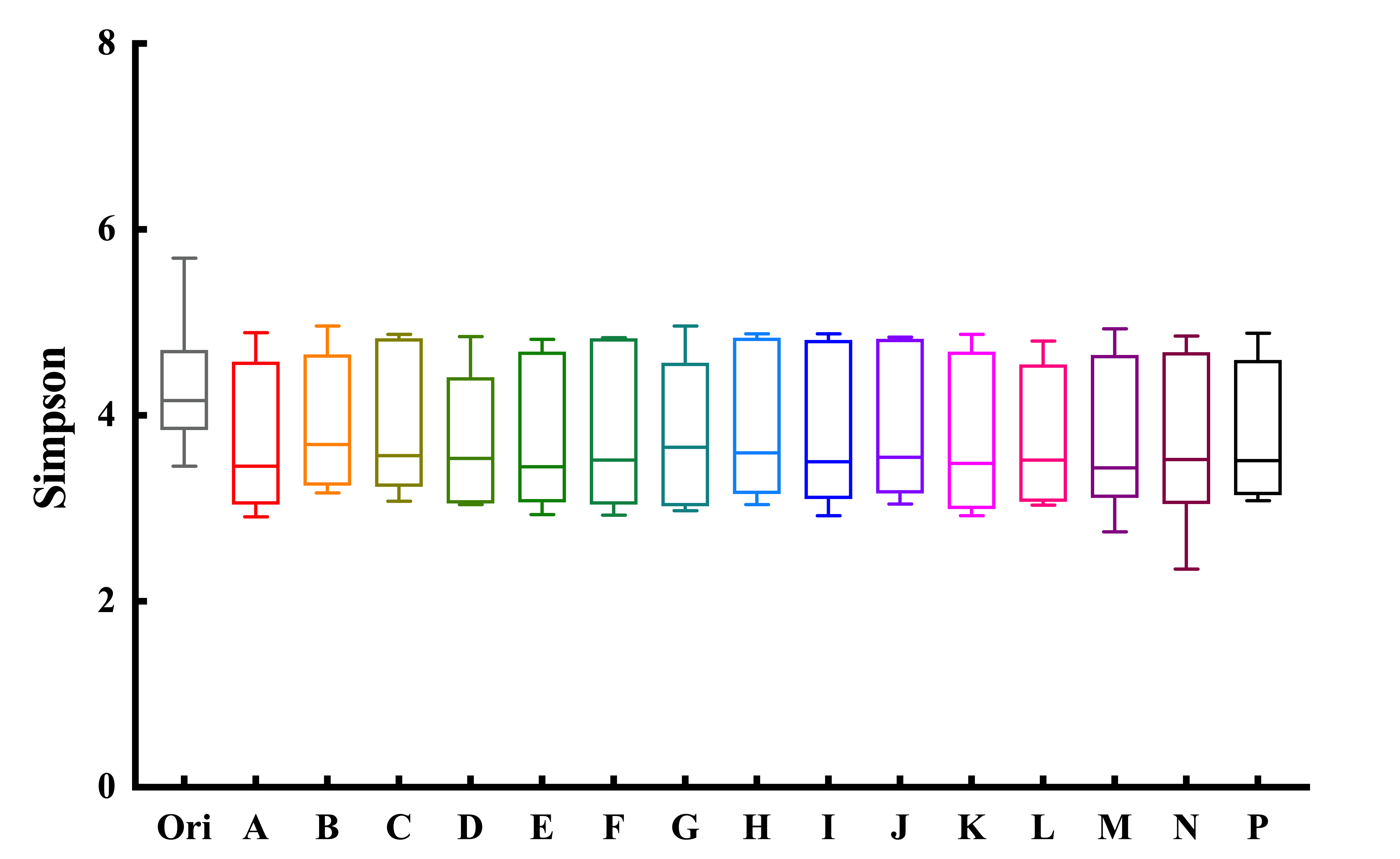

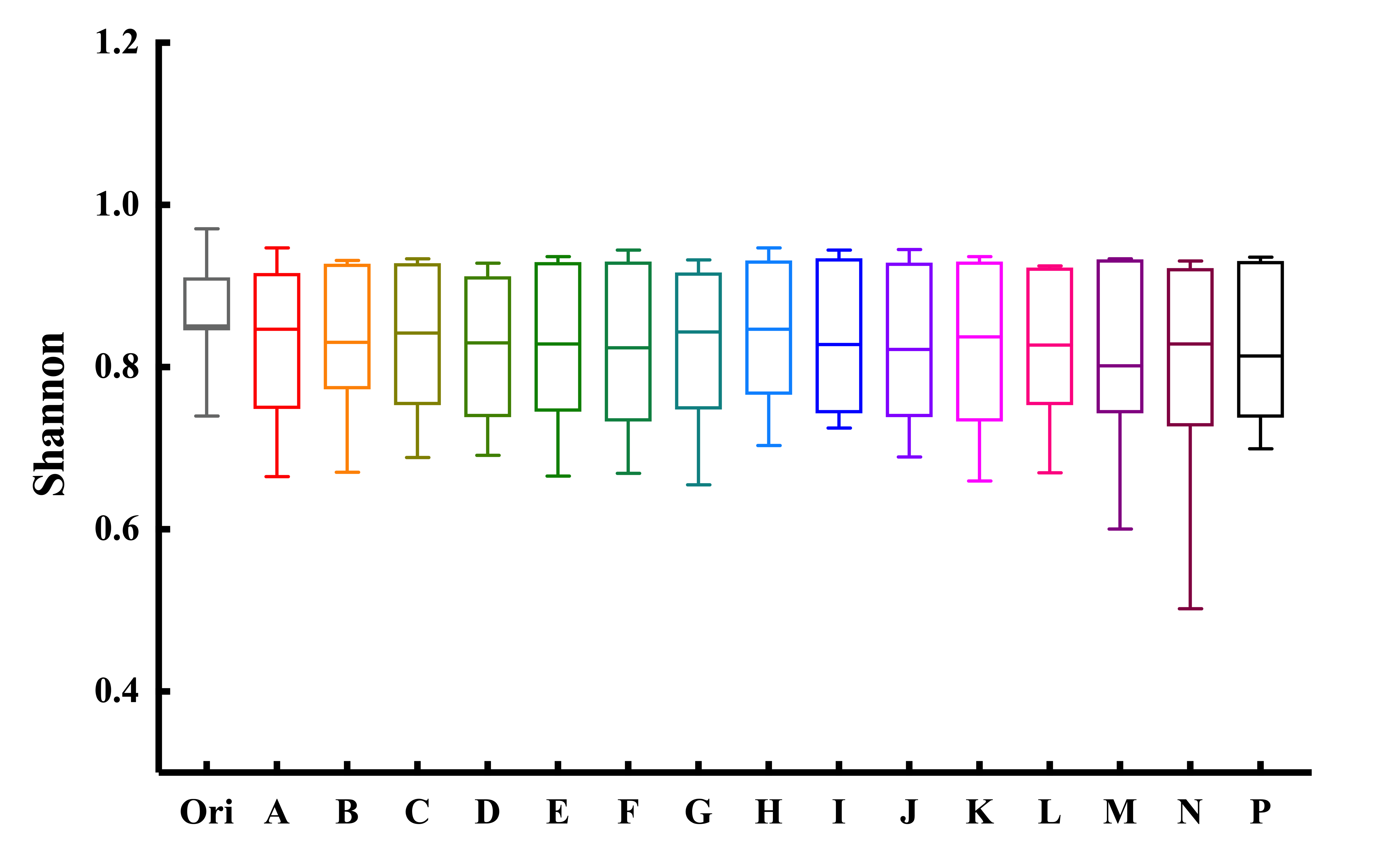
**

(C) (D)

**Supplementary Figure 1.** Alpha diversity indices of fermented fecal samples at the operational taxonomic unit (OTU) level. (A) Ace indice, (B) Chao indice, (C) Simpson indice, and (D) Shannon indice. Data are expressed as mean ± SD (n = 7). Note: A, satsuma orange; B, chachiensis; C, ponkan; D, lane late navel orange; E, blood orange; F, apple pomelo; G, majia pomelo; H, grapefruit; I, dekopon; J, fertile orange; K, lemon; L, sichuan kumquat; M, longyan kumquat; N, bergamot; P, blank control; Ori, fecal sample.
